# The impact and implementation of an mHealth intervention to improve infant and young child feeding in Senegal: IIMAANJE protocol for a cluster randomized control trial

**DOI:** 10.3389/fpubh.2023.1258963

**Published:** 2023-09-25

**Authors:** Shauna M. Downs, Daouda Gueye, Medoune Sall, Bamba Ndoye, Ndèye Ndambao Sarr, Moussa Sarr, Souleymane Mboup, Neeloy Ashraful Alam, Adama Diouf, Emily V. Merchant, Joachim Sackey

**Affiliations:** ^1^Department of Health Behavior, Society and Policy, School of Public Health, Rutgers University, New Brunswick, NJ, United States; ^2^Institut de Recherche en Santé, de Surveillance Epidémiologique et de Formation (IRESSEF), Pole Urbain de Diamniadio, Dakar, Senegal; ^3^Consulting and Training Group, Dakar, Senegal; ^4^Laboratoire de Recherche en Nutrition et Alimentation Humaine (LARNAH), Faculté des Sciences et Techniques, Université Cheikh Anta Diop, Dakar, Senegal; ^5^Sydney School of Public Health, The University of Sydney, Sydney, NSW, Australia; ^6^School of Health Professions, Rutgers University, Newark, NJ, United States

**Keywords:** Africa, behavior change communications, diets, infant and young child feeding (IYCF), mobile health, nutrition, cluster randomized control trial

## Abstract

Behavior change communication (BCC) strategies have the potential to improve infant feeding and nutrition outcomes among infants and young children in low- and middle-income countries. More recently, there has been a shift toward the adoption of mHealth interventions—the use of mobile phones to transmit health-related information or direct care—to promote recommended BCC strategies among the caregivers of infants and young children. In Senegal, most infants and young children are not fed according to recommended practices leading to a high prevalence of undernutrition and micronutrient deficiencies. The aims of this cluster randomized control trial, using an effectiveness-implementation (type 1) hybrid design, were to: (1) determine the impact of an mHealth IYCF intervention on IYCF practices and nutrition outcomes; and (2) examine the implementation, costs, and opportunities for scaling up the mHealth messaging intervention. The trial was conducted in three regions in Senegal (Thies, Fatick, Diourbel) with 488 mother, father and children (6–23 months) triads. The intervention included 8 scripted messages, that underwent cognitive testing prior to the intervention implementation, and 8 unscripted messages from positive deviants. One voice message and one text message were sent each week to members of our experimental group for a 16-week period. The impact of the intervention was assessed through a household survey, 24-h dietary recall, and hemoglobin measurements before and after the intervention implementation. The primary outcomes were minimal acceptable diet (MAD) and anemia. We also included a total of 54 participants in nine focus groups held with mothers and fathers and semi-structured interviews with Badienou Gox (i.e., community health workers) (*n* = 6) and national partners and program implementers (*n* = 6) to examine the intervention implementation process. The study was registered prior to data collection on Clinicaltrials.gov (Identifier: NCT05374837).

## Introduction

It is estimated that between 691 and 783 million people experienced hunger in 2022 (1). Moreover, 29.6% of the population is moderately or severely food insecure, 22% of children under five are stunted, and 6.8% are wasted globally (1). In West Africa, the prevalence of moderate to severe food insecurity is more than double the global average (1). Undernutrition is the number one risk factor for morbidity and mortality in Senegal, a country in West Africa with a large population, and disproportionately affects infants and young children (2).

The first 1,000 days, from conception to a child's second birthday, is a critical window to intervene to prevent malnutrition (3). During this time period, inadequate nutrition is associated with poor physical growth and cognitive development, reduced educational attainment and economic productivity, as well as increased risk for obesity and diet-related non-communicable diseases (4, 5). The use of appropriate, evidence-based infant and young child feeding (IYCF) practices, including breastfeeding and complementary feeding from 6–23 months, is essential for preventing malnutrition (6–8). Growth faltering and micronutrient deficiencies peak during the 6–23 month period, coinciding with the introduction of complementary foods (9). Only 10% of Senegalese children 6–23 months are fed according to IYCF recommendations, resulting in extremely high rates of anemia (71%) as well as a high prevalence of stunting (18 %) (10, 11). Improving complementary feeding has the potential to prevent malnutrition among young children in the first 1,000 days.

Behavior change communication (BCC) strategies aimed at increasing caregiver knowledge and addressing cultural norms that impede optimal feeding practices are effective in improving IYCF practices, particularly in several countries in Asia and Africa (12–14). BCC strategies go beyond traditional nutrition education by providing the reasoning for the importance of adopting specific behaviors, targeting actions to operationalize the messaging, and placing a strong emphasis on behavior change through the cultural lens of the target population. Unfortunately, BCC strategies often fail to reach caregivers of young children. Community Health Workers (CHWs) and Badienou Gox (meaning “community grandmothers” in Wolof) tasked with delivering IYCF counseling to mothers in Senegal are often limited by time and geographical constraints as well as competing health priorities (15). Identifying feasible, effective, and scale-able IYCF interventions, and alternative delivery platforms, is critical to improving IYCF practices in order to reduce the burden of malnutrition.

Mobile health (mHealth) messaging interventions, which utilize short messaging service (e.g., text messages), wireless data transmission, voice calling, and smartphone applications to transmit health-related information or direct care (16), have shown potential to alter health behaviors in high-income countries. However, there is limited literature on the impact of mHealth IYCF interventions in low-to-middle income countries (17). Given the high penetration of mobile phones in Senegal (18–20), even among rural populations, there is immense potential for the use of mobile technology to overcome the gaps in access to IYCF counseling and to broaden the reach of BCC interventions that promote appropriate, evidence-based IYCF practices. Well-designed and tailored mHealth interventions have shown some promise in improving IYCF practices, but research on their impact, implementation, and costs is limited (12). Moreover, mHealth IYCF interventions have yet to be scaled-up in Senegal, despite the urgent need to improve IYCF practices.

Given the high rates of child malnutrition among Senegalese children, interventions aimed at improving IYCF practices have the potential to lead to substantial gains in both health and productivity. Using mHealth delivery platforms could accelerate the dissemination of knowledge related to recommended IYCF practices and complement the existing activities of community health workers and Badienou Gox. While our previous 4-week mHealth IYCF voice messaging intervention pilot conducted with a small sample (*n* = 47) of households showed a 19.2% increase in minimum acceptable diet (MAD) (an indicator of dietary diversity) in children 6–23 months (14), a more rigorous evaluation is needed. Building on our formative research (14), we designed a user-centered mHealth IYCF voice messaging intervention based on the theory of planned behavior (21). The intervention targeted both mothers and fathers of young children (6–23 months) in Senegal to improve their IYCF knowledge and to address community norms that lead to suboptimal feeding practices. The aims of the effectiveness-implementation hybrid (type 1) cluster randomized control trial (cRCT) were to (a) determine the impact of an mHealth IYCF messaging intervention on IYCF practices, MAD and anemia, and (b) examine the implementation, costs and opportunities for scaling-up the mHealth IYCF messaging intervention in households with children 6–23 months in three rural villages in Senegal.

## Methods and analysis

This manuscript describes the protocol for the “*Impact de l'Intervention Mobile pour l'Amélioration de l'Alimentation auprès des Nourrissons et Jeunes Enfants (IIMAANJE)*” study. IIMAANJE is a cluster randomized control trial to improve health outcomes among 6–23 month old children in Senegal. Thus far, the recruitment, dissemination, and data collection for this study have been conducted. The data analysis and process evaluation are forthcoming.

### Setting

**Figure 1:**
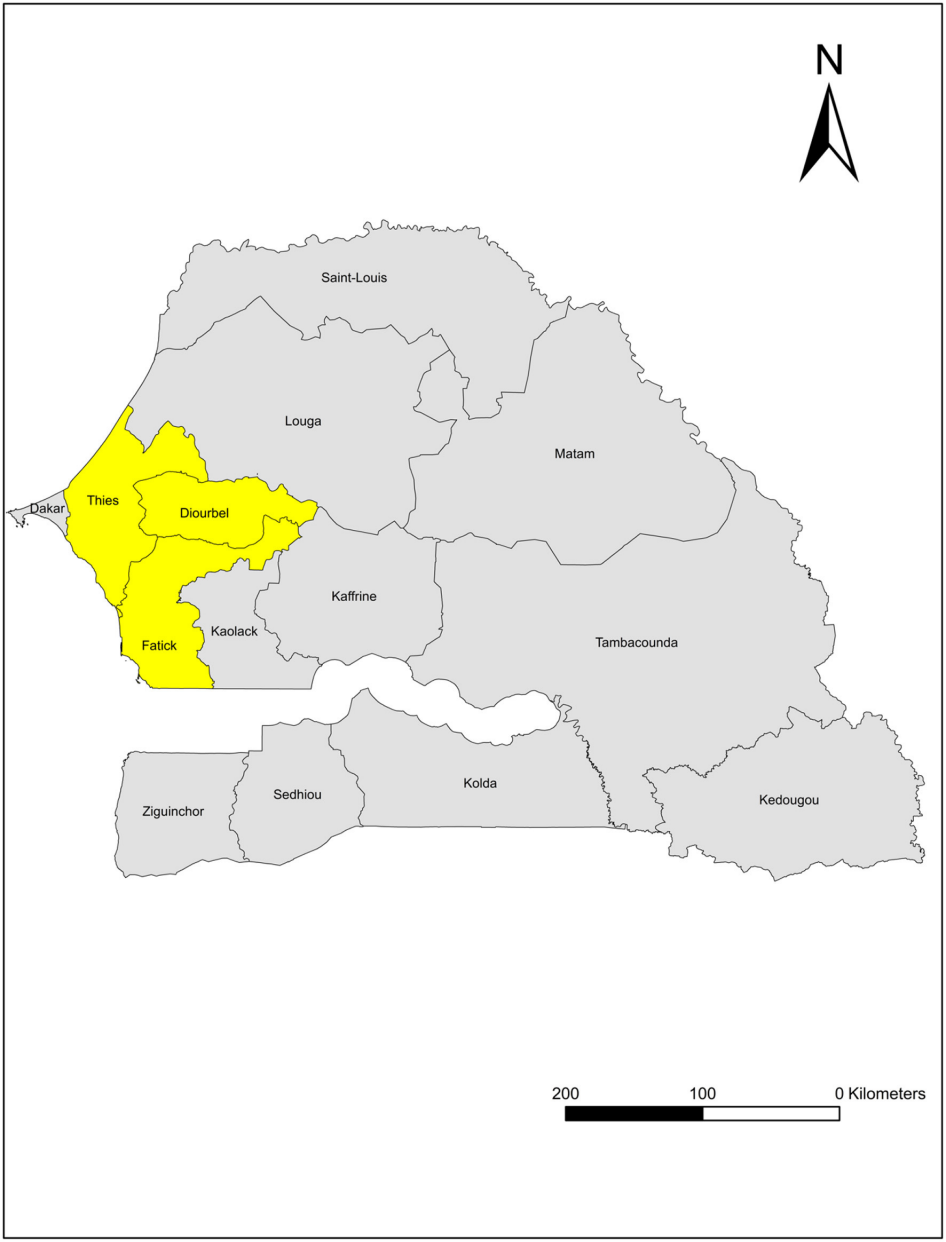
A map of the study regions in Senegal. Yellow color denotes the study regions: Thiès, Diourbel, and Fatick.

This study was conducted in 3 regions in western Senegal (Thiès, Diourbel, and Fatick). Senegal, located in West Africa, has a Sahelian, hot and semi-arid, climate with one rainy season from approximately June to September. Figure 1 provides a map of our study regions in Senegal. The study regions form the groundnut belt of Senegal and include both coastal and inland areas.

The coastal influence and worsening effects of climate change have resulted in significant seasonal variability, season to season and year to year, which impact agricultural production (22). The primary source of livelihoods in these communities is subsistence farming. Farming households are disproportionately affected by high rates of malnutrition and poverty (60%) (23). Moreover, our previous work found a high prevalence of anemia (66%), suboptimal feeding practices, and inadequate diets among children 6–23 months in these regions (Thiès, Diourbel, and Fatick) (unpublished data). The current study built on previous work by implementing a voice messaging intervention in those same communities and examining its impact. We conducted the study with existing farm groups in 104 villages within the aforementioned regions of Senegal.

### Design

**Figure 2:**
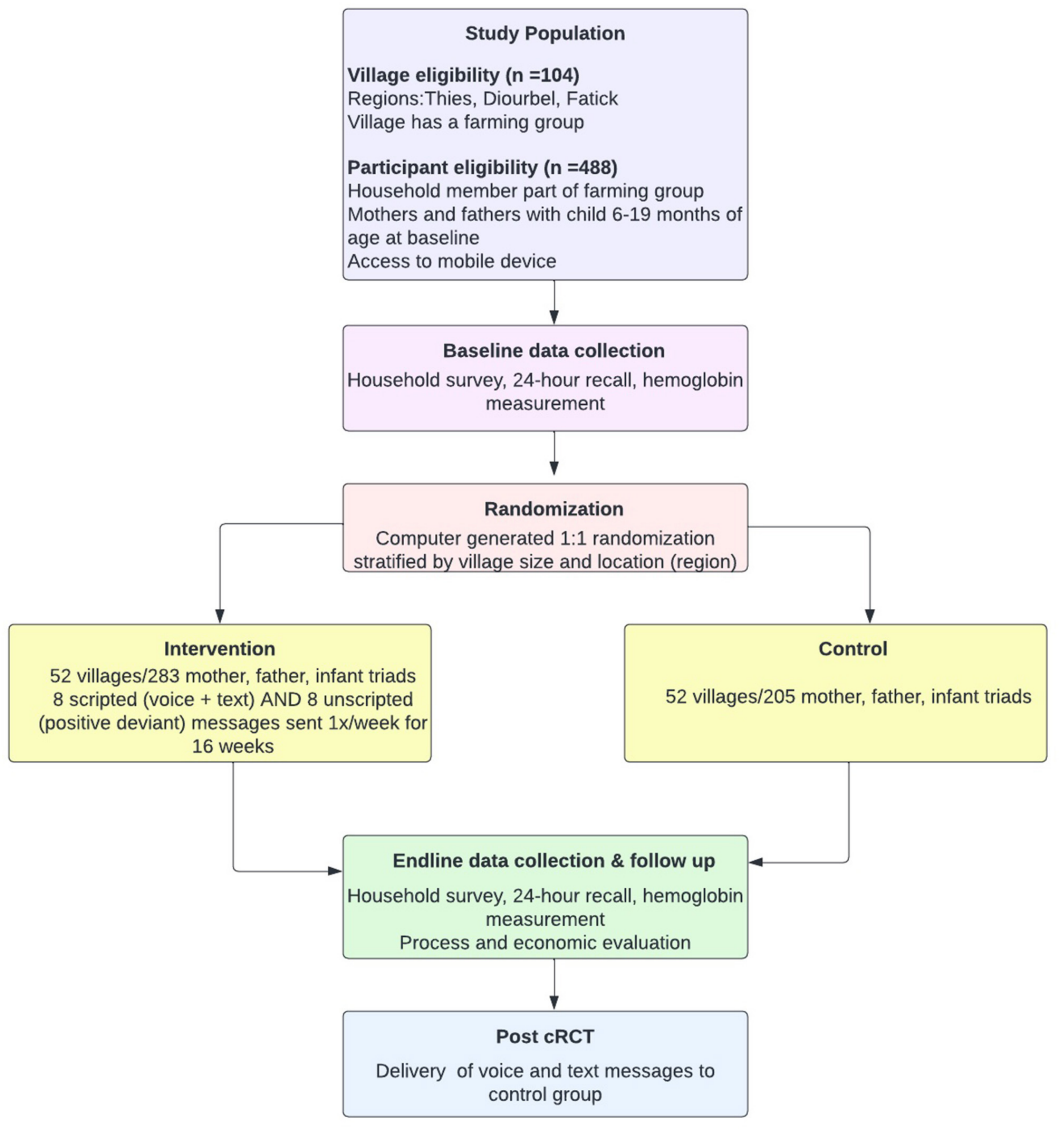
An overview of the study approach.

The overarching design of this study was an effectiveness-implementation hybrid (type 1) design using mixed-methods to test the intervention's impact while simultaneously gathering implementation data, allowing for improved translation of research findings (24, 25). The intervention impact was assessed using a cRCT. The implementation was assessed through three modes: (1) collection of process data throughout the intervention delivery; (2) in post-intervention household surveys; (3) and through specify semi-structured interviews and focus group discussions after the completion of the intervention. Figure 2 provides an overview of the study design.

Our sample size estimation was calculated based on our two primary outcomes: the prevalence of MAD and anemia. The sample size estimate was informed by our prior cross-sectional research on over 1200 household surveys and our pilot data over a 4-week intervention period (unpublished data) (14). Attrition was 2%, anemia prevalence was 66% and MAD prevalence was 46.8%. Assuming a conservative intra-class correlation coefficient of 0.02 (26), 5% attrition (based on our pilot work and previously published community-based research projects in Senegal) (27, 28), and a 2-sided significance level of 0.05, 104 clusters with 5 triads per cluster would provide 81.7% power to detect a 6% absolute difference in anemia and 88.8% power to detect an absolute difference of 7% in MAD. Our pilot study found a 19.2% increase in MAD. Even if we assume 10% attrition, we would still have 80% power to detect a 6% absolute difference in anemia and 87.3% power to detect an absolute difference of 7% in MAD. Improvements in anemia and MAD prevalence of 6 and 7%, respectively are clinically relevant and aligned with improvements in previously published studies (26, 29). In some instances, the number of participants in larger villages was increased given that some villages did not have a sufficient number of children in the target age group.

Ethical approval for this study was obtained from Rutgers University and the Comité National d'Ethique pour la Recherche en Santé (CNERS). We also obtained administrative authorization and letters of introduction from the Ministry of Health and Social Actions (MHSA). The study was registered prior to data collection on Clinicaltrials.gov (Identifier: NCT05374837). Given the low-risk nature of this trial, we did not have a Data Monitoring and Safety Board.

#### Cluster randomized control trial

Computer-generated random numbers were used to randomize 104 villages in a 1:1 allocation, stratified by village size (number of households per village) and region (Thiès, Diourbel, Fatick), to receive either the intervention (experimental group) or usual care (control group). Randomization was conducted following the baseline survey and given the nature of the intervention, was not blinded. The intervention was delivered over a 16-week period, which falls within the duration observed in previously published IYCF messaging interventions that demonstrated positive impacts (12). One voice and one text message were sent each week over the 16-week period, which is aligned with both the previously published literature (12) as well as the community preferences identified through our formative work. Using a combination of voice and text messages enabled us to ensure that messages reach phones in areas with poor mobile coverage (by sending text messages), while also accounting for literacy levels (by sending voice messages). Baseline and endline surveys were conducted in both the control and experimental groups. The control group received the 16 messages (2 messages per week) after the completion of endline data collection.

#### Process evaluation

Collecting implementation data is an essential component of impact evaluations (30), given that the potential effectiveness of interventions can be reduced by as much as 50% due to contextual factors that interfere with implementation (31). Without gaining a deeper understanding of why, how, for whom, and in which contexts the intervention works (or does not work), there can be missed opportunities for scale-up (31). Furthermore, existing literature related to the implementation and costs of IYCF interventions in LMICs is scarce (12, 32).

The process evaluation for this study was guided by a previously published framework to assess intervention implementation and developed specifically for cRCTs (33). This framework allowed us to assess the intervention's fidelity and quality of implementation, reach, and contextual factors associated with variations in outcomes, and clarified causal pathways (31, 33, 34). The process evaluation data will be used for both (i) providing feedback to keep the program “on track” (formative use), and (ii) to interpret and explain intervention outcomes (summative use) (35). The process evaluation is currently ongoing.

### Selection of study participants

#### Cluster randomized control trial

To inform our sampling for the cRCT, we conducted a pre-baseline survey in March-April 2022 to obtain a full census of all the households in each of the farming groups from our study villages as well as information about whether the household had children that would be eligible to participate in the study. As part of our previous work in this setting, we had already generated lists of households from the local farming groups. However, given that there were changes to the compositions of households in the farming groups, we updated these lists in the pre-baseline survey. We subsequently used the updated lists to identify eligible households for inclusion in the trial based on them having a child that was 6–19 months at baseline to ensure that they did not surpass 23 months at endline. From those lists, we randomly selected approximately five households (mother, father, child triads) per village to be included in the trial. Given that there were fewer than five households in some of our study villages with a child in our target age range, we had a smaller number of triads from some villages. The specific inclusion criteria for this study included being a mother, father, or primary caregiver of a child 6–19 months at the time of the baseline survey and have access to a mobile phone. In some cases, women included in the study did not have a mobile phone of their own but had access to the phone of the household head or another member of the household. Participation in our prior pilot study did not influence households' recruitment for this cRCT.

To recruit trial participants, we approached households that had been randomly selected to be included in the study, described the study, and ascertained their written consent to participate. If a household that had been randomized to our study sample chose to not participate in the study, we then recruited from the next household on our list of “reserve” households.

Mothers and fathers (or male and female primary caregivers) of children (6–19 months at baseline) living in households that were part of local farming groups were included in the trial. In some cases, the primary caregiver of the child may be a close relative rather than the birth mother. In these situations, the primary caregiver and her spouse or principal male caregiver both received the voice messages.

#### Process evaluation

We collected process data from all mothers and fathers in the experimental group using a combination of data captured by the voice messaging platform and endline surveys. We conducted semi-structured interviews with project implementation staff to generate data on implementation and operation-related challenges (data forthcoming). In addition, we purposively selected nine villages to collect more in-depth qualitative data related to the implementation process using focus groups with mothers and fathers in the experimental group as well as individual semi-structured interviews with each village's Badienou Gox (i.e., community health workers). The 9 villages were purposively selected to maximize variation in characteristics such as region, size, distance to urban settings, distance to markets, and ethnic and language diversity. Within each of the 9 villages, all participants in the intervention group were invited to participate in the focus groups. Lastly, we purposively selected a small number (~2–3) of program implementers as well as key stakeholders working in IYCF program uptake to participate in interviews.

### Description of intervention

We delivered a 16-week mobile voice messaging intervention that aimed to improve IYCF practices to mothers and fathers of young children. A total of 16 messages, half of which were scripted and the other half which were unscripted were delivered to mothers and fathers. The messages were sent in Wolof, based on the preferences of the study participants.

The eight scripted voice messages were developed based on our formative research. The voice messaging was based on the key messages booklets for infant and young child feeding from UNICEF and CNDN “*Conseil National de Développement de la Nutrition”* (National Council for Nutrition Development), ensuring that the messaging was consistent with both global and local recommendations. The theoretical underpinning of the way the messages are scripted is the Theory of Planned Behavior (TPB) (36). Using the TPB as the basis for the message framing, which places importance on the caregiver's beliefs about the behavior, their perceived efficacy of performing the behavior and the perceived benefits of performing the behavior (36), we developed and piloted 8 primary messages related to: breastfeeding until 2 years of age, consuming a variety of nutritious foods within a given meal and avoid providing energy-dense foods of low nutritional quality, preparing a thicker consistency of porridge to provide more nutrients, the importance of animal sourced foods, consuming vitamin A rich fruits and vegetables, consuming leafy greens, handwashing, and feeding infants and young children fruits and vegetables produced and foraged by the household (see Supplementary Table 1 for messages). We had several local experts in IYCF review the messages prior to their finalization.

Based on our pilot data, some participants had difficulty understanding some of the messages. We therefore conducted cognitive testing of the scripted messages prior to implementing the intervention. Cognitive testing is a form of qualitative research that allows interviewers to probe for a deep understanding of comprehension by asking participants to paraphrase the messages, discuss their associated thoughts and emotions, and offer suggestions for improvements (37). This increases the likelihood that the information delivered in the messages would be interpreted by its recipients in the way in which it was intended. To cognitively test the interviews, we conducted 3 focus groups in each of our 3 study regions. We used this approach to overcome any linguistic divides that might exist between the three selected regions.

In addition to the eight scripted messages, eight unscripted messages were developed from “positive deviants” in the pilot villages. Positive deviance is a behavior change approach based on the observation that even in low-resource communities there are people who deviate toward preferred practices (“positive deviants”) (38). These individuals have uncommon, but successful, strategies that enable them to find better solutions than their peer (38). Including role models in IYCF interventions can increase their effectiveness (39–41). We identified “positive deviants” whose children were thriving from a growth and nutrition perspective in the pilot villages where the cognitive testing was completed. These villages are culturally, linguistically, socially, and economically similar to the villages included in our cRCT.

Local Badienou Gox helped our study team identify the positive deviants given their familiarity with the children in their community and their role in assessing their growth and nutritional status. We recorded unscripted messages of the personal experiences of these role models that were used to reinforce the 8 primary messages based on the TPB. In total, we recorded messages from 5 women who were approached for in-depth interviews to learn more about their personal experience implementing IYCF practices. The interviews were recorded, transcribed in Wolof, and used to develop the messages. The cognitive testing took place in the pilot villages between February-May 2022.

We used a Senegalese owned and operated company (OuA company; https://ouacompany.com) to send the text and voice messages. The voice messages were sent at the day/time of week that participants indicated a preference for during the baseline surveys. If the recipient did not answer the phone when they received the initial voice message call, the message delivery platform attempted to call them one additional time.

### Data collection

#### Cluster randomized control trial

##### Primary and secondary outcomes

The primary outcomes for this study included: the prevalence of minimal acceptable diet (MAD) and anemia as well as changes in MAD and anemia prevalence from baseline to endline. Secondary outcomes included: minimum dietary diversity (MDD), minimum meal frequency (MMF), the frequency of consuming the foods targeted in the intervention using a food frequency questionnaire (FFQ) over the past 7 days, IYCF practices using the UNICEF IYCF indicators (42), and IYCF knowledge, attitudes, norms and intentions of both mothers and fathers. Table 1 provides a more detailed description of the study outcomes.

**Table 1:** An overview of the study outcomes measured at baseline and endline.

**Outcome type**	**Outcome**	**Description**
Primary	Anemia prevalence	We used Hemocue Hb301 machines to measure hemoglobin levels in children to determine anemia prevalence using the WHO cut-offs: mild 10 ≤ hb < 11 g/dl; moderate 7 ≤ hb < 10 d/dl and severe hb < 7 g/dl (43). A finger prick was used to obtain a drop of capillary blood that was placed on a cuvette and inserted in the Hemocue machine to obtain an on-the-spot assessment of hemoglobin levels.
	Minimum Acceptable Diet	The 24-h recall was used to calculate minimum acceptable diet (MAD) a composite indicator that draws from minimum dietary diversity (MDD) and minimum meal frequency (MMF). Children who meet the thresholds for both MDD and MMF (defined below) are defined as consuming a MAD, based on the UNICEF IYCF indicator (42).
Secondary	Minimum Dietary Diversity	The 24-h recall was used to calculate minimum dietary diversity (MDD) (consuming 5 of 8 food groups (breast milk; grains, roots and tubers; pulses, nuts, and seeds; dairy products; flesh foods; eggs; vitamin A rich fruits and vegetables; other fruit and vegetables) (44).
	Minimum Meal Frequency	The 24-h recall was used to calculate minimum meal frequency (MMF) (solid, semi-solid or soft foods at least 2x/day of for breastfed infants 6–8 months; 3x/day for breastfed children 9–23 months; and 4x/day for non-breastfed children 6–23 months, where meal options also include milk and at least one meal is solid, semi-solid, or soft).
	Frequency of consuming key foods in past 7 days	We used a 7-day food frequency questionnaire (FFQ) to assess the frequency that children consumed specific foods targeted in the intervention over the course of the previous week. More specifically, the FFQ ascertained the number of times that the following foods were consumed: eggs, leafy greens, fish, milk, cowpea, nuts, orange-colored fruits and vegetables, other fruits and vegetables, thick porridge, beef or mutton, pork, chicken, and liver.
	IYCF practices	We used the UNICEF IYCF indicators to assess feeding practices. Mothers were asked about feeding practices as part of the household surveys. These included: Bottle feeding 0–23 months; Continued breastfeeding 12–23 months; Exclusive breastfeeding under 6 months; Exclusively breastfed for the first 2 days after birth; Egg and/or flesh food consumption 6–23 months; Early initiation of breastfeeding; Ever breastfed; Introduction of solid, semi-solid or soft foods 6–8 months; Minimum dietary diversity 6–23 months; Mixed milk feeding under 6 months; Minimum meal frequency 6–23 months; Minimum milk feeding frequency for non-breastfed children 6–23 months; Sweet beverage consumption 6–23 months; Unhealthy food consumption 6–23 months; Zero vegetable or fruit consumption 6–23 months (42). The proportion of children being fed according to the detailed descriptions of these indicators was assessed based on the UNICEF IYCF indicator manual (42).
	IYCF knowledge, attitudes, norms and intentions	IYCF knowledge, attitudes, norms and intentions was assessed using survey questions based on the components of the intervention. Both mothers and fathers were asked the survey questions as part of the household survey. The questions were grounded in the theory of planned behavior and based on previously published IYCF knowledge, attitudes, norms and intentions questions by Monterrosa et al. (45). The questions were pilot tested by the project PI.

##### Data collection approach

In one household visit, we recruited and conducted a household survey using a list-based 24-h dietary recall (24HR) to examine the consumption of MDD, MMF and MAD among the children in our study. We also measured hemoglobin to assess anemia prevalence. Detailed descriptions of each of these is below. Data were collected in experimental and control groups, at baseline and endline, using electronic tablets.

*Household surveys*: The household surveys ascertained the following information: demographics, household characteristics and assets, household expenditures (including healthcare and food expenditures), household food security, the health of the child, and IYCF knowledge, attitudes, belies, norms, intentions, and practices. Both mothers and fathers separately answered the IYCF knowledge, attitudes, beliefs, norms and intentions survey questions, which were previously published and used in this population during our pilot (14, 45). The questions, informed by the TPB, examined pathways that lead to behavior change. Given that mothers are the primary caregivers of young children, we asked them to report the frequency of consuming specific foods targeted in the intervention using a FFQ as well as a list-based 24HR, which are the most appropriate ways of collecting dietary data for infants and young children (46–48). In addition, mothers were asked questions about: their child's consumption of iron-rich or iron-fortified foods, the introduction of solid, semi-solid or soft foods, and breastfeeding practices that will be used to assess IYCF practices using the validated UNICEF IYCF indicators (42), which are associated with growth outcomes in children (49).

*List-based 24-h dietary recall*: A list-based 24-h dietary recall was administered with mothers to assess the dietary intakes of their children. This assessed whether their child consumed a list of foods and beverages (including breast milk) over the past 24 h. The list based 24-h recall was administered according to the UNICEF IYCF indicators guidance document (42). As part of the household survey, the enumerator would first explain that they were going to read out a list of food groups and that the respondent should indicate which groups were consumed by the infant or young child during the previous day and night. Prior to data collection, we created a comprehensive list of commonly consumed foods in each of the food groups to increase the ease and accuracy of data collection. Supplementary material B provide the list of food groups. Based on the dietary recall we calculated MAD, which measures the proportion of children aged 6–23 months who: had meal frequency the previous day that met the minimum standard for their age as defined by the WHO; AND consumed foods from at least 5 of the 8 food groups identified by the indicator.

*Hemoglobin measurement*: Hemocue Hb301 machines were used to measure hemoglobin in children in order to determine anemia prevalence using the WHO cut-offs: mild 10 ≤ hb < 11 g/dl; moderate 7 ≤ hb < 10 g/dl; and severe hb < 7 g/dl (43). Hemocue machines allow for non-invasive point-of-care assessments of hemoglobin levels using capillary blood from a finger prick. Standard procedures for their operation were used based on the protocol used by the Senegal DHS (10). While a majority of anemia may be due to iron deficiency related to suboptimal dietary intakes, some may be due to other causes including malaria (50, 51).

#### Process and economic evaluation

##### Data collection approach

###### Process evaluation questions post message delivery

After each voice message was sent, participants were called on the phone to ask a series of yes/no questions related to the content of the message. More specifically, they were asked: if they understood the message, if they found the message helpful, and whether they intended to adopt the behavior. OuA recorded these responses in an excel file.

###### Tracking of call logs for voice message delivery

The OuA messaging platform was used to track whether the voice messaging calls were answered and if so, how long they were listened to. The platform captured these data for all the mothers and fathers enrolled in the experimental group for each of the messages. This information was used to assess the reach of the intervention.

###### Endline survey process evaluation questions

We included a series of questions related to the intervention implementation, as part of the endline survey conducted after its implementation. Mothers and fathers in the experimental group were asked to recall (unprompted) the messages that were sent as part of the messaging intervention. We also included questions related to program satisfaction including whether they enjoyed receiving the messages, whether they understood them, if they were delivered at an appropriate frequency, their confidence in their ability to adopt the behaviors mentioned in the messages and whether they believed that the messages led to improvements in their IYCF practices. These questions had already been field tested as part of the piloting of this intervention (14).

###### Semi-structured interviews/focus groups

The focus group discussions were conducted with mothers and fathers (*n* total = 54) in the experimental group of the nine villages where the process evaluation focus groups were conducted. The focus group discussion guide was designed to capture the intervention's fidelity, dose delivered and received as well as contextual information that may have influenced the intervention implementation process. The Badienou Gox who facilitated the implementation of the intervention in each village were also invited for an in-depth semi-structured interview. They worked as voluntary outreach community workers to support the selection, mobilization, and follow-up of the study participants in selected villages. In each of the nine villages, the Badiene Gox was interviewed to capture their perceptions of the potential impacts of the intervention, any constraints faced during its implementation or barriers that community members may have faced in adopting the behaviors described in the messages, their satisfaction with the intervention as well as their perceptions of the community's satisfaction. We are also conducting semi-structured interviews with members of the OuA team to gain additional understanding of the intervention implementation, including any challenges experienced as well as potential key stakeholders related to the intervention's scale up. All focus groups and interviews were conducted, recorded, and translated (from Wolof to English) and transcribed verbatim. In each village, mothers and fathers who participated in the interventions were invited to participate in the focus groups.

###### Economic evaluation

The intervention costs were documented throughout the duration of the intervention. Average costs per child in the experimental group were estimated over the period of the intervention implementation. Healthcare utilization and costs were assessed in both the experimental and control groups at both baseline and endline. To ascertain healthcare costs, we asked mothers if they had taken their child to a health center (hospital, health post, etc.) over the past 4 months. The time frame of 4 months was selected given that it coincided with the duration of the intervention. If they indicated that they took their child to a health center they were asked about the costs associated with it, including transportation costs, consultation or hospital costs, laboratory costs, prescription costs, and any other costs that they may have encountered due to the visit.

### Data analysis

#### Cluster randomized control trial

While we completed the data collection for this trial, the data analysis is forthcoming. To estimate the impact of the mHealth messaging intervention on anemia and MAD, as well as on our secondary outcomes, we plan to utilize a difference-in-differences approach. Logistic regression models will be fitted for each outcome, with explanatory variables that include the intervention indicator, time (baseline and endline), and intervention by time interaction. The interaction term is of primary interest, as it summarizes mean changes in the outcome before and after the intervention in the treatment group compared with the control group. The intervention variable is classified based on randomization (i.e., intention to treat). We anticipate that the parallel trend assumption will hold in this scenario since the various clusters and triads within clusters are similar to each other socio-economically; however, we will carefully test this assumption by applying the recently developed difference-in-difference model checklist (52). We will analyze the data on the individual level and use generalized estimating equation (GEE) extensions of regression in the statistical software package SAS to account for clustering. All models will adjust for sex of the child, socioeconomic status of the family and highest education completed by the mother. We will also conduct exploratory subgroup analyses based on baseline anemia status.

#### Process and economic evaluation

The process evaluation data analysis has not yet begun. We plan to use a combination of quantitative data captured through the messaging platform and endline survey as well as qualitative data from the focus groups discussions and semi-structured interviews for the analysis. Quantitative data will be analyzed using descriptive statistics. Qualitative data will be open-coded and analyzed according to key themes related to the intervention fidelity and quality of implementation, reach, and contextual factors influencing its delivery and uptake. These data will be collated with the quantitative data to summarize the implementation challenges and opportunities for scale-up in different contexts, guided by the process evaluation framework for cRCTs (33).

The economic evaluation of the intervention will be analyzed by assessing the total costs of the intervention, as well as average costs per child in the experimental group, estimated over the period of the intervention implementation. Where possible, a cost consequence analysis will be conducted that compares the average cost per child in the experimental and usual care group against the health consequences of both groups (53, 54).

## Discussion

The use of mobile technologies as part of BCC strategies has the potential to improve infant and young child feeding (IYCF) practices. Previous studies conducted in Sub-Saharan Africa have found that mHealth interventions can improve and reduce the cost of patient monitoring, adherence to medications, as well as healthcare worker communication, particularly in rural areas (16). However, there is a lack of evidence related to the effectiveness (including cost-effectiveness) of mHealth interventions to warrant its large-scale implementation. Evidence related to the impact of mHealth interventions on IYCF outcomes is even more scarce.

In Senegal, counseling mothers about IYCF is the responsibility of community health workers (CHW). However, their reach is limited due to time and geographical constraints as well as competing health priorities (55–57). The use of mHealth to complement the work of CHWs has a real potential to improve infant and young child nutrition and health outcomes. Some efforts have been made to seize the opportunity of using mobile technology, given the relatively high rates of mobile use in the country (58), to help address public health issues in Senegal, particularly in the field of nutrition and maternal health (59). However, pilot studies or interventions carried out provided little or no information on the effectiveness or impact of the use of mobile technologies (60). Given that there are many contextual challenges to scaling up interventions that leverage mobile technology to improve nutrition and health outcomes, rigorous evaluations of both the impact and implementation of mHealth IYCF interventions are needed.

This study was the first trial to examine the impact and implementation of an IYCF mHealth intervention in Senegal. The findings of this study will be used to inform future opportunities for integrating mHealth into existing programming as well as other opportunities for scale-up. We plan to disseminate the study findings to key stakeholders in Senegal. In addition, a summary of the study findings will be disseminated to the study participants using mobile technology.

## Conclusion

This article provides an overview of our study protocol which aims to examine the impact, implementation, and costs of an mHealth IYCF messaging intervention in Senegal using a hybrid effectiveness-implementation model (type 1). The study findings will help to inform best practices related to IYCF behavior change communication programs in Senegal targeting rural populations, and opportunities for scaling-up the mHealth IYCF messaging intervention.

## Ethics statement

Approval to conduct this trial was obtained from the Senegal Ministry of Health's National Ethics Committee (CNERS), and the Rutgers University IRB, New Jersey. Written consent was obtained from parents/guardians of young children to participate in this research. While we are including young children in this study, they are too young to provide assent.

## Author contributions

SD: Conceptualization, Funding acquisition, Methodology, Project administration, Writing—original draft, Writing—review and editing. DG: Funding acquisition, Methodology, Project administration, Supervision, Writing—review and editing. MSal: Conceptualization, Data curation, Methodology, Supervision, Writing—review and editing. BN: Writing—review and editing, Data curation, Formal analysis, Methodology, Software, Supervision. NNS: Investigation, Methodology, Supervision, Writing—review and editing. MSar: Funding acquisition, Writing—review and editing. SM: Funding acquisition, Writing—review and editing. NAA: Funding acquisition, Writing—review and editing. AD: Supervision, Writing—review and editing. EVM: Writing—review and editing. JS: Conceptualization, Data curation, Formal analysis, Funding acquisition, Methodology, Writing—original draft, Writing—review and editing.
