# Supplementary material for: The impact and implementation of an mHealth intervention to improve infant and young child feeding in Senegal: IIMAANJE protocol for a cluster randomized control trial
Source: Front Public Health. 2023 Sep 25;11:1258963. doi: 10.3389/fpubh.2023.1258963 (PMC10561905; doi:10.3389/fpubh.2023.1258963)
Supplement: Supplementary file 1 [file Table_1.DOCX]

Supplemental Table 1. Scripted and unscripted voice messages* delivered to mothers and father

| **Key Scripted Messages for Voice Messaging Intervention** | |
| --- | --- |
| **Key Message** | **Voice Message** |
| 1. **Continue breastfeeding until 2 years of age** | Breastmilk is the most complete and nutritious food for your baby. It provides the perfect balance of proteins, fats, vitamins, and minerals needed for optimal growth and development. Around 6 months of age, your baby may be ready for complementary foods like fish, cowpea, millet, fruits, and vegetables, which provide additional nutrients and help develop their taste preferences. It's important to continue breastfeeding on demand both day and night until your baby is at least 2 years old. By combining breastmilk and healthy complementary foods, you can give your baby the best start in life and support their lifelong health and well-being*.* |
| 1. **Feed your baby a variety of foods in addition to breastmilk multiple times a day** | Hello sweet mother! Starting at about 6 months, your baby needs other foods in addition to breast milk to grow well**.** In addition to feeding your baby breastmilk, you should feed your baby a variety of foods. It's important to gradually introduce new foods to your baby. Some healthy food options for babies include melon, papaya, mango, and watermelon, as well as madd, tamarind, black prune, and sump. Try to offer a variety of foods at each meal to ensure your baby receives the necessary nutrients for optimal growth and development. |
| 1. **Feed your child thicker porridge consistency** | Your baby's stomach is small and therefore, feeding them runny porridge may not provide the necessary nutrients for optimal growth. Instead, it is recommended to prepare thicker porridge, such as lakh and fonde, and incorporate nutrient-rich foods such as peanuts, ground niebe, ground nebbeday, and cowpea. Ensure the porridge is thick enough to adhere to the spoon during feeding. This will provide your baby with the necessary energy and nutrients they require for healthy development. |
| 1. **Feed your child animal source foods** | Animal-source foods, such as liver, are crucial for proper infant growth and to prevent illnesses. As soon as your baby is 6 months, start feeding them animal source-foods. To help your baby grow well and get enough nutrients, feed your baby unsweetened lait caille [local yogurt] and tastes of fish, seafood, liver and eggs. Babies can eat well-cooked and finely-chopped fish with the bones removed, seafood and eggs even if they don’t have teeth. These foods can be digested well by your baby and, without them, it will be difficult for them to grow well. Every time you cook fish for your family, save a small piece for your baby. If you give these foods often you can help, make sure your baby grows well and prevent health issues like anemia. |
| 1. **Feed yellow or orange fruits and vegetables rich in Vitamin A at least 3 times per week** | Orange fruits and vegetables are rich in Vitamin A which is important for your baby’s eye health and the prevention of illnesses. At least 3 times per week, feed your baby yellow or orange fruits or vegetables. For example, fruits like mango, papaya, and cantaloupe or foods such as cut carrots, orange sweet potato will help your baby be healthy. |
| 1. **Feed leafy greens every day** | To provide your baby with important nutrients feed them dark leafy greens every day. Manioc, nebedaya, Mbuum, potates and niebe [local examples] leaves have vitamins that help your baby’s growth and development and provide your baby with iron. Many nutritious leafy greens can be gathered from the wild. Dark leafy greens can be added to porridge or mashed up to make it easy for your baby to eat. To make it easier for the child to consume green leaves, you should grind them and add them to foods such as sauces and porridges that they consume. Wash these leaves well before feeding them to your baby. |
| 1. **Wash hands with soap and water regularly** | Good hygiene is important to avoid diarrhea and other illnesses. Unclean hands are often the cause of illness. Avoiding diarrhea and infections can help your baby grow better. Wash your hands with soap and water before preparing foods and feeding baby**.** Wash your hands and your baby’s hands before eating**.** Wash your hands with soap and water after using the toilet and washing or cleaning baby’s bottom. You should also wash your hands before breastfeeding, wash the bowls and other utensils used to feed the child and cover the food to protect them against dust and flies. The lack of good hygiene can make your child ill. |
| 1. **Feed your child some of the vegetables you grow and livestock you raise** | The vegetables and fruits that you grow can provide your child with important nutrients. Also, raising livestock, including poultry such as chickens, as well as goats, sheep, and other animals, not only provides a source of wholesome meat but also offers the opportunity for additional income. Although crops are an important source of income, leaving some in the household can make a big difference for your child’s health. Feeding your child, the vegetables that you grow can help them to get the nutrients they need to be healthy. |
| **Key Un-scripted Messages from positive deviants for Voice Messaging Intervention** | |
| **Key Message** | **Voice Message** |
| 1. **Feed your child with breastmilk, your main source of nutrition** | Greetings! My name is Adja Ndao, and my daughter Sokhna, has surpassed six months of age and she has been thriving since her birth. I nurse her at least 8 to 10 times a day and night. I have observed that breast milk fortifies her immune system and shields her from both diarrhea and respiratory infections. I prioritize breastfeeding as I have discerned that breast milk is conducive to her growth and well-being. Additionally, I have noted that the more Sokhna nurses, the greater my lactation and her alertness. Truly, there is nothing that compares to breast milk for a baby! Nonetheless, it is essential to supplement your child's diet with other foods when she reaches six months of age. |
| 1. **Feed your child with solid foods to complement the breast milk** | Greetings, my name is Yacine, one of your sisters… Ever since my son Mohamed reached the age of six months, I have been providing him with purees or mashed vegetables twice a day, as well as porridge. Nonetheless, I continue to breastfeed him until he reaches the age of two. At six months of age, breast milk alone is no longer sufficient to guarantee your child's growth and well-being. Therefore, in addition to breast milk, I offer Mohamed a variety of nutrient-rich foods such as rice, colored vegetables, dark green foliage, seafood, meat, and eggs. I also use legumes such as cowpeas, beans, and peas, combined with vitamin-rich foods. These dietary practices have culminated in Baby Mohamed's thriving and advancing each day. |
| 1. **Your healthy hygiene routine protects your child against disease** | Good evening! My name is Dibor and my son is 12 months old. In addition to breastfeeding, I provide him with solid porridge and other foods from our family meals such as potatoes, sweet potatoes, fish, colorful vegetables (such as carrots, zucchini), dark green leaves (such as mboum), eggs, beans, and lentils. Apart from his three regular meals, I also give him one or two snacks each day. For his snacks, I prepare vegetable purees and fruits such as mango, papaya, and banana. Sometimes I also give him eggs, niebe, or peas. These dietary habits help him grow, stay healthy mashaAllah. |
| 1. **Continue breastfeeding when your child is feeling sick** | Halloo, I am Adja. I believe that children are highly susceptible to illnesses such as diarrhea and other infections. When my child falls ill, in addition to seeking medical attention, I increase hygiene, duration, and frequency of breastfeeding. I have noticed that breastfeeding helps my child recover faster and prevents weight loss. Illness often leads to loss of appetite in children, but I exercise patience and encourage my child to eat their favorite foods. Reorganizing nutritional practices for a sick child aids in a faster recovery. |
| 1. **Provide complementary foods after 6 months** | Greetings, my name is Soda! My son's name is Ablaye, and he is 8 months old. Ablaye fell very ill once, and after visiting the doctor, it was revealed that he had a vitamin deficiency. After being prescribed treatment, the doctor advised me to provide him with a diverse diet, rich in a variety of vitamins such as papaya, ripe mango, and other fruits with orange or yellow hues, as well as sweet potatoes with orange flesh, dark green vegetables, liver, and milk. Since then, I have been resolutely committed to providing him with a nutritious and varied diet. Thanks to this approach, Ablaye is now thriving, free from any illness or infection, and growing normally. A healthy, varied, and nutritious diet is crucial for a child's well-being! |
| 1. **Feed your child with a variety of foods to preserve their health** | Good day, I am Ramatoulaye. I regularly provide animal-source foods to my child, "Babacar mou ndaw," who is a little over a year old, and he is thriving. To maintain his strength, I prioritize products such as liver, red meat, and small fish, as well as colorful vegetables and dark green leaves to prevent anemia. Fruits with orange hues, such as ripe papaya, mango, and sweet potatoes with orange flesh, are also important. His dietary habits have proven successful, "mashaAllah." Additionally, I take him to health facilities for vaccination, vitamin A supplementation, deworming, and growth monitoring. He is free from illnesses such as anemia, diarrhea, infections, or vitamin deficiencies, and he is healthy, happy, and spends his time smiling and having fun. |
| 1. **Feed your child preserved leafy greens for their health and growth across seasons** | Good day, my name is Awa Mbaye, and I am a mother to a six-month-old child named Hawa. I prioritize the incorporation of green leaves such as "nebbeday" into my child's diet, and I urge other mothers to do the same. Green leaves are highly nutritious, and since my daughter Hawa started using them, she has been thriving. I frequently provide her with leaves such as "nebeday" or "sapp-sapp." To prepare them, I pick the leaves, wash them with bleach water, and dry them by spreading them out on a dryer or "INDER." I typically place the dryer in a shaded area to protect the leaves from the sun, which could deplete their essential nutrients. Once they are dry, I grind them into powder and store them in sachets. This enables me to fortify all the foods I give to Hawa with a powder rich in nutrients from green leaves. |
| 1. **Improve your child’s nutrition and health with fortified flour** | Greetings, my name is Henriette, and my child's name is Jean Pierre. He just turned 8 months old and is thriving in good health. To enhance his breastfeeding, I mainly provide him with porridge made from improved flour. I learned how to make this flour during a training provided by a local NGO. To prepare this improved flour for your child, you should first clean and rinse millet or other cereals, take a small portion, mix it with roasted niébé flour and nebeday powder, grind it with a milling machine, and store the resulting powder in small sachets to preserve its nutritional value. I use this powder most often to enhance Jean Pierre's diet. Improved flour is highly nutritious, protects my son against diseases, helps him grow better, and provides him with strength. |
