# Supplementary material for: The impact and implementation of an mHealth intervention to improve infant and young child feeding in Senegal: IIMAANJE protocol for a cluster randomized control trial
Source: Front Public Health. 2023 Sep 25;11:1258963. doi: 10.3389/fpubh.2023.1258963 (PMC10561905; doi:10.3389/fpubh.2023.1258963)
Supplement: Supplementary file 2 [file Table_2.DOCX]

**Supplemental Material A.**

Food and beverage groups included in list-based 24-hour recalls

| **Section 7. INFANT YOUNG CHILD FEEDING PRACTICES: LIQUIDS QUESTIONNAIRE** | | | |  |
| --- | --- | --- | --- | --- |
| Now I would like to ask you about liquids that [NAME] had yesterday during the day or at night.  Please tell me about all drinks, whether [NAME] had them at home, or somewhere  else. | | | |  |
|  | Yesterday during the day or at night, did [NAME] have…? | |  | |
| 7.1 | Plain water | Plain water? | 1= Yes  2= No | |
| 7.2 | Formula | Infant formula, such as Similac? | 1= Yes  If “yes”: How many times did [NAME] drink  formula? _______  2= No | |
| 7.3 | Animal milk | Milk from animals, such as fresh, tinned or  powdered milk? | 1= Yes  If “yes”: How many times did [NAME] drink milk from animals? _______  If “yes”: Was the milk or were any of the milk  drinks a sweet or flavoured type of milk?  2= No | |
| 7.4 | Yogurt drinks | Yogurt drinks such as lait caille? | 1= Yes  If “yes”: How many times did [NAME] drink yogurt? _______  If “yes”: Was the yogurt or were any of the  yogurt drinks a sweet or flavoured type of  yogurt drink?  2= No | |
| 7.5 | Chocolate flavored | Chocolate-flavoured drinks including  those made from syrups or powders? | 1= Yes  2= No | |
| 7.6 | Fruit juice | Fruit juice or fruit-flavoured drinks  including those made from syrups or  powders? | 1= Yes  2= No | |
| 7.7 | Soda | Sodas, malt drinks, sports drinks or energy  drinks? | 1= Yes  2= No | |
| 7.8 | Tea | Tea, coffee, or herbal drinks? | 1= Yes  If “yes”: Was the drink/ Were any of these  drinks sweetened?  2= No | |
| 7.9 | Broth | Clear broth or clear soup? | 1= Yes  2= No | |
| 7.10 | Other liquids | Any other liquids? | 1= Yes  If “yes”: what was the liquid or what were  the liquids?  If “yes”: Was the drink or were any of these  drinks sweetened?  2= No | |

| **Section 8. INFANT YOUNG CHILD FEEDING PRACTICES: FOODS QUESTIONNAIRE (24-HOUR RECALL)** | | | | |
| --- | --- | --- | --- | --- |
| Now I would like to ask you about foods that [NAME] had yesterday during the day or at night.  I am interested in foods your child ate whether at home or somewhere else. Please think about snacks and small meals as well as main meals. I will ask you about different types of foods, and I would like to know whether your child ate the food even if it was combined with other foods in a mixed dish like tiboudienne.  Please do not answer “yes” for any food or ingredient used in a small amount to add flavour to a dish. Yesterday during the day or at night, did [NAME] eat: | | | | |
| 8.1 | Yogurt | Yogurt, other than yogurt drinks? | 1= Yes  If “yes”: How many times did [NAME] eat yogurt?  2= No |  |
| 8.2 | Grain | Porridge, bread, rice, noodles, pasta, couscous, sorghum, etc.? |  |  |
| 8.3 | Orange veg | Pumpkin, carrots, sweet red peppers, squash or  sweet potatoes that are yellow or orange inside? |  |  |
| 8.4 | Root veg | Plantains, white potatoes, white yams, manioc, cassava  or turnip? |  |  |
| 8.5 | Leafy green | Dark green leafy vegetables, such as baobab greens, cow pea greens, bisssap leaves, etc.? |  |  |
| 8.6 | Other veg | Any other vegetables, such as eggplant, green pepper, bitter melon, okra, tomato, etc? |  |  |
| 8.7 | Orange fruit | Ripe mangoes or ripe papayas or red palm fruit pulp? |  |  |
| 8.8 | Other fruit | Any other fruits, such as apples, oranges, watermelon, etc.? |  |  |
| 8.9 | Organ meat | Liver, kidney, heart or gizzards? |  |  |
| 8.10 | Processed meat | Sausages, hot dogs/frankfurters, ham, bacon, salami,  canned meat, etc.? |  |  |
| 8.11 | Meat | Any other meat, such as beef, pork, lamb, goat, chicken, duck, etc.? |  |  |
| 8.12 | Eggs | Eggs? |  |  |
| 8.13 | Shellfish | Fresh or dried fish or shellfish? |  |  |
| 8.14 | Pulses | Beans, peas, lentils, nuts , seeds, groundnut or cowpea, etc? |  |  |
| 8.15 | Cheese | Hard or soft cheese? |  |  |
| 8.16 | Sweets | Sweet foods such as chocolates, candies, pastries, cakes,  biscuits, or frozen treats like ice cream and popsicles,  or doughnuts? |  |  |
| 8.17 | Savory | Chips, crisps, puffs, French fries, fried dough, instant  Noodles, etc.? |  |  |
| 8.18 | Other solids | Any other solid, semi-solid or soft food? | If “yes”: What was the food? |  |
| 8.19 | Eat solids | Did [NAME] eat any solid, semi-solid or soft food  yesterday during the day or at night? | If “yes” probe: What kind of solid, semi-solid or soft  foods did [NAME] eat?  [mark food group]  *****Only ask if did not report eating any of foods above*** |  |
| 8.20 | Frequency solids | How many times did [NAME] eat any solid, semi-solid or  soft foods yesterday during the day or night? |  |  |
